# Draft Genome Sequence of *Cupriavidus* sp. Strain SK-3, a 4-Chlorobiphenyl- and 4-Clorobenzoic Acid-Degrading Bacterium

**DOI:** 10.1128/genomeA.00664-14

**Published:** 2014-07-03

**Authors:** Claudia Vilo, Michael J. Benedik, Matthew Ilori, Qunfeng Dong

**Affiliations:** a Department of Biological Sciences, University of North Texas, Denton, Texas, USA; b Department of Computer Science and Engineering, University of North Texas, Denton, Texas, USA; c Department of Biology, Texas A&M University, College Station, Texas, USA; d Department of Microbiology, University of Lagos, Lagos, Nigeria

## Abstract

We report the draft genome sequence of *Cupriavidus* sp. strain SK-3, which can use 4-chlorobiphenyl and 4-clorobenzoic acid as the sole carbon source for growth. The draft genome sequence allowed the study of the polychlorinated biphenyl degradation mechanism and the recharacterization of the strain SK-3 as a *Cupriavidus* species.

## GENOME ANNOUNCEMENT

Polychlorinated biphenyls (PCB) are pollutants that are difficult to remove from contaminated sites due to their low capacity for degradation and their bioaccumulation in the environment (1, 2). The *Cupriavidus* sp. strain SK-3, originally isolated from PCB-contaminated tertiary lagoon sludge, grows on both 4-chlorobiphenyl (4-CB) and 4-clorobenzoic acid (4-CBA) as the sole carbon source (1, 2). However, no specific enzymes have been characterized for its metabolic capacity (3).

The SK-3 genome was sequenced via Eureka Genomics (Hercules, CA) by using the Illumina genome analyzer IIx from paired-end libraries with an average insert size of 221 bp and read length of 51 bp. The total number of reads was 10,024,280, with a total length of 511,238,280 bp (about 53-fold coverage of the estimated genome size). We followed an improved assembly strategy recently published by Soueidan et al. (4). Briefly, after adapter trimming and quality filtering with the TrimGalore software (Barbraham Boinformatics) (http://www.bioinformatics.babraham.ac.uk/projects/trim_galore/), the Mix program (4) was applied to combine the assembly results from three different genome assemblers, SOAPdenovo2 (5), ABYSS (6), and MaSuRCA (7). Mix software removes low-quality and redundant contigs, identifies overlapping contigs, and merges overlapping contigs into longer ones. In total, this strategy produced 299 final scaffolds as our final assembly. The Rapid Annotation using Subsystem Technology (RAST) server version 4.0 (8) was used for genome annotation.

The SK-3 assembled draft genome was 7,429,145 bp long, with a GC content of 65%, and had 6,834 protein-coding genes, which are similar to those of other sequenced *Cupriavidus* species (9–11). Based on the RAST annotation, the SK-3 genome showed an enrichment of genes related to PCB degradation, such as those for biphenyl-degrading enzymes and dioxygenases (12). Although a previous study classified SK-3 as a *Burkholderia* sp. based on biochemical results (1), our analysis based on the 16S rRNA gene phylogeny strongly showed that SK-3 belongs to the *Cupriavidus* genus, as SK-3 is tightly clustered with *Cupriavidus* bacteria and clearly separated from *Ralstonia* and *Burkholderia* bacteria on the phylogenetic tree. Previous studies of several biphenyl-degrading bacteria have shown that the *bph* operon is involved in PCB degradation (3, 12). We have identified for the first time the existence of the *bph* operon genes in the SK-3 strain, which are in the order of *bphI-bphJ-orfx1-orfx2-bphH-orfx3-bphB-bphA2-bphA1-bphA3-orfx4-orfx5-bphD*. Interestingly, a clear difference in the phylogeny of the *bph* operon genes exists between SK-3 and *Cupriavidus* sp. strain SK-4, which was isolated together with SK-3 from the same PCB-contaminated tertiary lagoon sludge (1, 2, 13). *Cupriavidus* sp. strain SK-4 can use ortho-substituted CB congeners as a sole carbon source (1, 13, 14), and it also grows on all monochlorobiphenyls, as well as some dichlorobiphenyls, such as 2,2′-dichlorobiphenyl and 2,4′-dichlorobiphenyl (1, 14). The SK-3 *bph* genes are more similar to those in *Sphingobium yanoikuyae* B1, while the SK-4 *bph* operon genes are almost identical to those in *Burkholderia xenovorans* LB400 (13, 14). In addition, the genes in the SK-4 *bph* operon are in a rearranged order compared to those in SK-3 (14). These phylogenetic differences indicate that SK-3 and SK-4 acquired their *bph* operons via independent horizontal gene transfer events and may also account for their preferences on different PCB substrates.

### Nucleotide sequence accession numbers.

The draft genome sequence of strain SK-3 has been deposited at DDBJ/EMBL/GenBank under the accession number JFJV00000000. The version described in this paper is the second version, JFJV02000000.
