# Erratum for Vilo et al., Draft Genome Sequence of *Cupriavidus* sp. Strain SK-3, a 4-Chlorobiphenyl- and 4-Chlorobenzoic Acid-Degrading Bacterium

**DOI:** 10.1128/genomeA.00756-14

**Published:** 2014-07-31

**Authors:** Claudia Vilo, Michael J. Benedik, Matthew Ilori, Qunfeng Dong

**Affiliations:** a Department of Biological Sciences, University of North Texas, Denton, Texas, USA; b Department of Computer Science and Engineering, University of North Texas, Denton, Texas, USA; c Department of Biology, Texas A&M University, College Station, Texas, USA; d Department of Microbiology, University of Lagos, Lagos, Nigeria

## ERRATUM

Volume 2, no. 4, e00664-14, 2014. Page 1: The article title should read as given above and the word “clorobenzoic” should read “chlorobenzoic” in the abstract and main text.

